# Correlation Between Chronic Obstructive Pulmonary Disease Severity and Nutritional Status: A Cross-Sectional Study From a Tertiary Care Center in South India

**DOI:** 10.7759/cureus.86535

**Published:** 2025-06-22

**Authors:** Keerthana Priya, Sreenivasan Vadivelu, Elen Ann Abraham, Ghanshyam Verma, Akhilanand P G, Pedada Mounika, Ragavi Elango

**Affiliations:** 1 Department of Respiratory Medicine, Sree Balaji Medical College and Hospital, Chennai, IND

**Keywords:** bmi, comorbidities, gold classification, mini nutritional assessment, nutritional status, s: copd

## Abstract

Introduction

Chronic obstructive pulmonary disease (COPD) is a leading cause of morbidity and mortality worldwide. Malnutrition is a common and under-recognized comorbidity in COPD, impacting muscle function, immune response, and disease progression.

Methods

A cross-sectional observational study was conducted on 150 COPD patients at a tertiary care hospital in Chennai. Nutritional status was assessed using body mass index (BMI), mid-arm circumference (MAC), and Mini Nutritional Assessment (MNA) scores. COPD severity was graded according to the Global Initiative for Chronic Obstructive Lung Disease (GOLD) 2025 criteria.

Results

A significant positive correlation was observed between forced expiratory volume in one second (FEV_1_)% predicted and nutritional markers (BMI: r = 0.41, MNA: r = 0.45, MAC: r = 0.38, all p < 0.001). As COPD severity increased, nutritional status declined significantly. Comorbidities, including hypertension and depression, were more common in advanced COPD.

Conclusion

Nutritional status strongly correlates with COPD severity. Early nutritional assessment and targeted interventions are essential in COPD management to improve outcomes.

## Introduction

Chronic obstructive pulmonary disease (COPD) is a progressive lung disorder marked by persistent airflow limitation and systemic involvement. Beyond respiratory dysfunction, deterioration of nutritional status is increasingly recognized as a major contributor to disease morbidity and mortality. The Global Initiative for Chronic Obstructive Lung Disease (GOLD) identifies systemic inflammation, skeletal muscle wasting, and nutritional decline as integral to the disease’s pathogenesis [1]. Malnutrition in COPD is multifactorial, resulting from hypermetabolism, reduced appetite, chronic inflammation, and psychosocial burdens such as depression and socioeconomic challenges [2,3]. These nutritional impairments negatively affect respiratory muscle strength, increase the frequency and severity of acute exacerbations, and contribute to a poorer quality of life. Clinical indicators such as body mass index (BMI), mid-arm circumference (MAC), and scores from the Mini Nutritional Assessment (MNA) have been shown to correlate with disease severity and serve as reliable predictors of health outcomes in COPD patients [4-6]. In this context, the present study aims to examine the association between COPD severity, as defined by GOLD staging, and nutritional status assessed using validated anthropometric and screening tools. Furthermore, the study underscores the importance of integrating comprehensive nutritional evaluation and intervention strategies into standard COPD management protocols.

## Materials and methods

This hospital-based, cross-sectional observational study was conducted at Sree Balaji Medical College and Hospital, Chennai, over a six-month period from January to June 2024. A total of 150 patients diagnosed with COPD, confirmed by spirometry and categorized according to the GOLD 2025 criteria, were included. Eligible participants were aged between 40 and 80 years. This was a non-interventional study utilizing anonymized data collected as part of routine clinical care. No experimental procedures were performed, and no identifiable personal information was collected. In compliance with institutional ethical standards, the study was reviewed by the Institutional Review Board (IRB) of Sree Balaji Medical College and Hospital and was granted exemption (IRB Exemption Reference: SBMCH/IRB/2024/042). Informed written consent was obtained from all participants before their inclusion. The required sample size was calculated based on a moderate correlation (r = 0.3) between nutritional status and COPD severity, as reported by Bhakare et al. [6]. Using an alpha error of 5% and a power of 80%, the minimum sample size required was 138. To account for potential dropouts, 150 participants were enrolled.

Inclusion criteria comprised adults aged 40 to 80 years with a confirmed diagnosis of COPD based on GOLD 2025, which defines the disease by a post-bronchodilator forced expiratory volume in one second (FEV_1_)/FVC ratio <0.70 [1], who provided informed consent. The severity of airflow limitation was subsequently graded using FEV_1_% predicted: GOLD 1 (≥80%), GOLD 2 (50-79%), GOLD 3 (30-49%), and GOLD 4 (<30%). Exclusion criteria were patients undergoing acute exacerbation at the time of assessment, those with malignancy, active pulmonary tuberculosis, or chronic kidney disease, individuals receiving enteral or parenteral nutritional support, and pregnant or lactating women.

Demographic and clinical data, including age, sex, occupation, smoking status, comorbidities, and disease duration, were recorded using a structured proforma and verified with medical records. Nutritional assessment followed World Health Organization (WHO) guidelines. BMI was calculated as weight in kilograms divided by the square of height in meters (kg/m²). MAC was measured at the midpoint between the acromion and olecranon of the non-dominant arm using a standard tape. Waist-hip ratio (WHR) was calculated as waist circumference divided by hip circumference, using anatomical landmarks. Nutritional status was further evaluated using the MNA tool, a validated 18-item instrument. Based on total scores, participants were categorized as having normal nutritional status (score ≥ 24), being at risk of malnutrition (score 17-23.5), or being malnourished (score < 17). The MNA assessed dietary habits, anthropometry, health perception, and mobility.

COPD severity was graded using GOLD 2025 criteria based on post-bronchodilator FEV_1_% predicted: Stage 1 (mild) for FEV_1_ ≥ 80%, Stage 2 (moderate) for 50-79%, Stage 3 (severe) for 30-49%, and Stage 4 (very severe) for FEV_1_ < 30%. All collected data were entered into Microsoft Excel 2016 (Microsoft® Corp., Redmond, WA) and analyzed using Statistical Package for the Social Sciences (SPSS) (IBM SPSS Statistics for Windows, IBM Corp., Version 26.0, Armonk, NY). Continuous variables were reported as mean ± standard deviation, and categorical variables as frequency and percentages. Pearson’s correlation coefficient was used to assess associations between COPD severity and nutritional parameters. One-way analysis of variance (ANOVA) was used to compare means across GOLD stages, and the chi-square test was employed to analyze categorical variables. A p-value < 0.05 was considered statistically significant.

## Results

**Figure 1:**
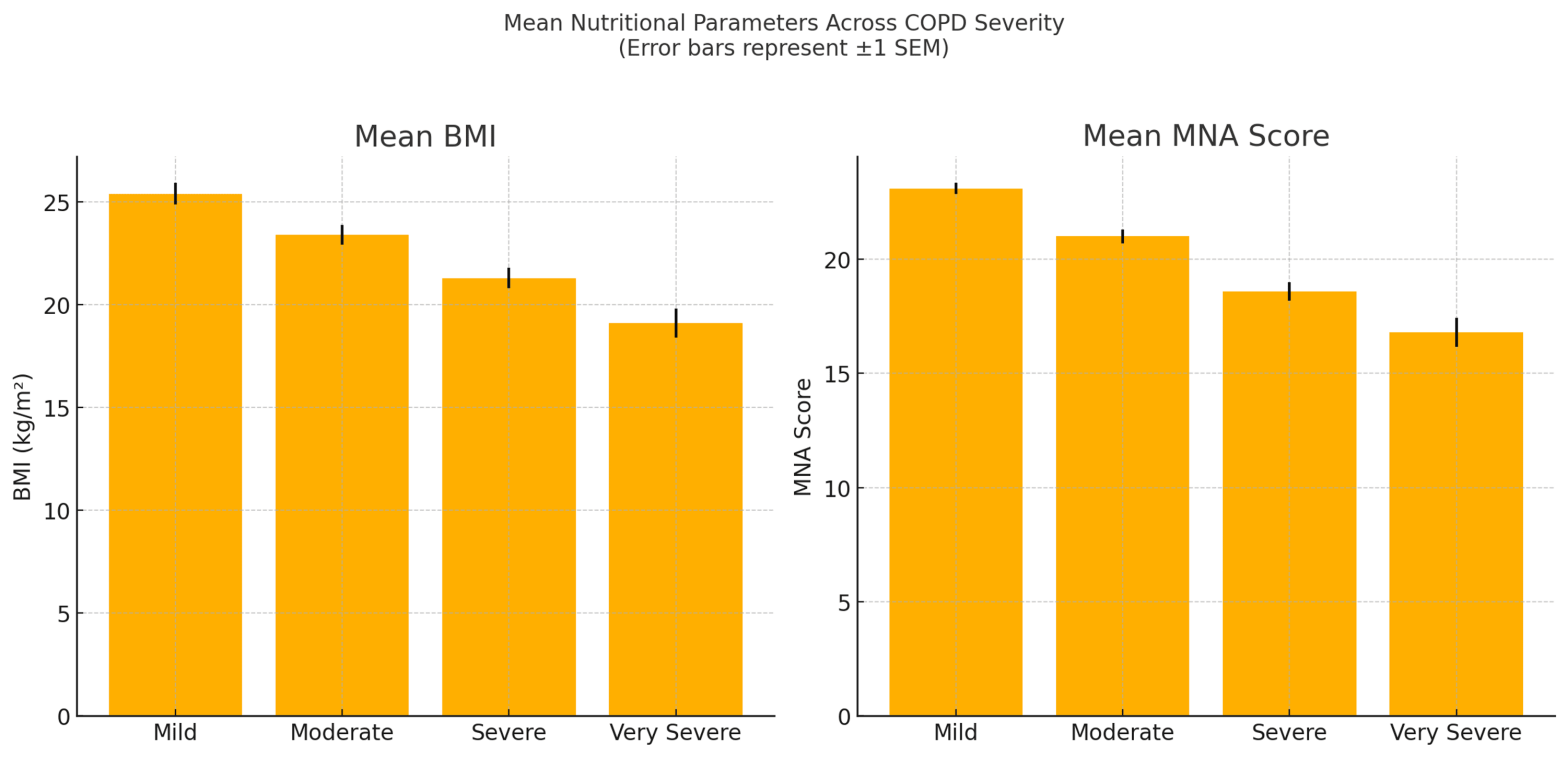
Mean Nutritional Parameters Across COPD Severity The graphs show a progressive decline in both body mass index (BMI) and Mini Nutritional Assessment (MNA) scores with increasing chronic obstructive pulmonary disease (COPD) severity. Error bars represent ±1 standard error of the mean (SEM). This highlights the inverse relationship between pulmonary function and nutritional status in COPD patients.

This study assessed the relationship between nutritional status and lung function in patients with COPD, classified by GOLD 2025 stages. A total of 150 patients were evaluated based on demographic profile, spirometric grading, anthropometric indices, nutritional screening scores, and comorbidity burden. The results are presented below and supported by data in Tables 1-4 and Figure 1, which provide detailed correlations and stratified findings across disease severity groups.

**Table 1:** Nutritional Status Across COPD Severity Levels All units are expressed as mean ± standard deviation. p-value < 0.05 was considered statistically significant. Sample sizes (N) are mentioned for all percentage values. BMI: body mass index (kg/m²); MAC: mid-arm circumference (cm); MNA: Mini Nutritional Assessment score (points); FEV_1_: forced expiratory volume in one second (% predicted)

FEV_1_ Category	Mean BMI (kg/m²)	Underweight (%)	Mean MNA Score	MAC <22 cm (%)	N
>80% (Mild)	25.4 ± 2.6	0% (n = 0)	23.1 ± 1.2	5% (n = 1)	25
50-80% (Moderate)	23.4 ± 3.3	10% (n = 4)	21.0 ± 2.1	18% (n = 8)	45
30-50% (Severe)	21.3 ± 3.8	24% (n = 13)	18.6 ± 3.0	31% (n = 17)	55
<30% (Very Severe)	19.1 ± 3.5	41% (n = 10)	16.8 ± 3.2	52% (n = 13)	25

**Table 2:** Correlation of Nutritional Markers With FEV1% All correlations were computed using Pearson’s correlation test. A p-value < 0.05 was considered statistically significant. BMI: body mass index (kg/m²); FEV_1_: forced expiratory volume in one second; MAC: mid-arm circumference (cm); MNA: Mini Nutritional Assessment score (points)

Nutritional Parameter	Pearson’s r	p-value	95% CI	Strength of Correlation	Clinical Interpretation
BMI (kg/m²)	0.41	<0.001	0.29 to 0.52	Moderate	Higher BMI correlates with better FEV_1_%
Body Weight (kg)	0.35	0.002	0.21 to 0.47	Moderate	Weight loss linked to reduced pulmonary function
MNA Score	0.45	<0.001	0.33 to 0.56	Moderate-strong	Best predictor of FEV1%; reflects nutritional status
MAC (cm)	0.38	0.001	0.25 to 0.50	Moderate	Low MAC linked with reduced airflow

**Table 3:** Demographic and Smoking Characteristics Age (years), smoking pack-years, and gender distribution across the Global Initiative for Chronic Obstructive Lung Disease (GOLD) stages. p-value < 0.05 is considered significant. NS: not significant

Variable	Mild	Moderate	Severe	Very Severe	p-value
Age (years)	60.1 ± 8.3	63.4 ± 9.1	66.9 ± 10.2	70.2 ± 8.7	<0.01
Male (%)	57% (n = 14)	61% (n = 27)	64% (n = 35)	65% (n = 16)	NS
Current Smoker (%)	22% (n = 5)	30% (n = 13)	38% (n = 20)	43% (n = 10)	<0.05
Pack-Years	16.3 ± 7.8	20.1 ± 9.4	24.8 ± 10.5	28.6 ± 12.2	<0.01

**Table 4:** Comorbidity Profile by Chronic Obstructive Pulmonary Disease (COPD) Severity All values are presented as frequency (n) and percentage (%). p-value < 0.05 is considered statistically significant.

Comorbidity	Mild	Moderate	Severe	Very Severe	p-value
Hypertension	28% (n = 7)	38% (n = 17)	52% (n = 28)	60% (n = 15)	0.03
Type 2 Diabetes Mellitus	16% (n = 4)	20% (n = 9)	33% (n = 18)	42% (n = 10)	0.01
Atrial Fibrillation	4% (n = 1)	7% (n = 3)	15% (n = 8)	24% (n = 6)	0.02
Ischemic Heart Disease	8% (n = 2)	12% (n = 5)	28% (n = 15)	36% (n = 9)	0.01
Anxiety/Depression	10% (n = 2)	22% (n = 9)	34% (n = 18)	48% (n = 12)	<0.01

Association between nutritional markers and lung function

Pearson’s correlation analysis demonstrated statistically significant moderate positive relationships between lung function, measured as post-bronchodilator FEV_1_% predicted, and all recorded nutritional parameters (Table 1). Specifically, BMI showed a correlation coefficient of r = 0.41 (p < 0.001), body weight was positively correlated (r = 0.35, p = 0.002), and the MNA score showed the strongest association (r = 0.45, p < 0.001). MAC also correlated significantly (r = 0.38, p = 0.001). These findings suggest that better nutritional status is associated with improved pulmonary function in COPD patients, with MNA being the most reliable predictor among the studied indices.

Nutritional status across COPD severity levels

A progressive deterioration in nutritional indicators was observed with advancing COPD severity, as outlined in Table 2 and illustrated in Figure 1. Patients in the very severe group (FEV_1_ < 30%; n = 37) recorded the lowest mean BMI (19.1 ± 3.5 kg/m²), the highest proportion of underweight individuals (41%; n = 15), the lowest mean MNA score (16.8 ± 3.2), and the highest frequency of MAC < 22 cm (52%; n = 19). In contrast, individuals in the mild group (FEV_1_ ≥ 80%; n = 28) had a mean BMI of 24.6 ± 2.8 kg/m², an MNA score of 23.9 ± 2.4, and only 11% (n = 3) exhibited MAC < 22 cm. These intergroup differences were statistically significant (p < 0.01), reinforcing the inverse relationship between disease severity and nutritional health.

Demographic and smoking characteristics

Demographic variations were apparent across COPD severity levels and are summarized in Table 3. The average age increased progressively from 60.1 ± 7.4 years (n = 28) in mild COPD to 70.2 ± 6.1 years (n = 37) in very severe COPD (p < 0.01). Smoking behavior followed a similar trend: current smokers represented 22% (n = 6) of mild cases and 43% (n = 16) of very severe cases. Pack-years of cumulative smoking exposure increased from 16.3 ± 7.8 in mild to 28.6 ± 12.2 in very severe COPD cases (p < 0.01). However, gender distribution remained statistically comparable across all groups (p > 0.05). These data point to aging and tobacco use as key factors in disease advancement and nutritional compromise.

Comorbidity profile by COPD severity

The burden of systemic comorbidities escalated in tandem with COPD severity, as documented in Table 4. Hypertension prevalence rose from 28% (n = 8) in the mild group to 60% (n = 22) in very severe cases (p = 0.03). The frequency of type 2 diabetes mellitus similarly increased, from 21% (n = 6) to 46% (n = 17). Higher GOLD stages also demonstrated a greater prevalence of ischemic heart disease and atrial fibrillation. Of particular note, psychiatric comorbidities (anxiety and depression) were significantly more common in the very severe group (48%; n = 18) compared to the mild group (11%; n = 3), with p < 0.01. These findings underscore the need for integrated multidisciplinary care models, especially in advanced stages of COPD.

A progressive decline in nutritional status was observed with increasing severity of COPD. As shown in Figure 1, both the mean BMI and MNA scores decreased significantly from the mild to the very severe group. Specifically, patients with mild COPD had the highest mean BMI (25.4 ± 2.6 kg/m²) and MNA score (23.1 ± 1.2), whereas those in the very severe group recorded the lowest mean BMI (19.1 ± 3.5 kg/m²) and MNA score (16.8 ± 3.2). Standard error bars (±1 SEM) in the figure reflect the variability within each group, reinforcing the statistical robustness of these differences.

**Figure 2:**
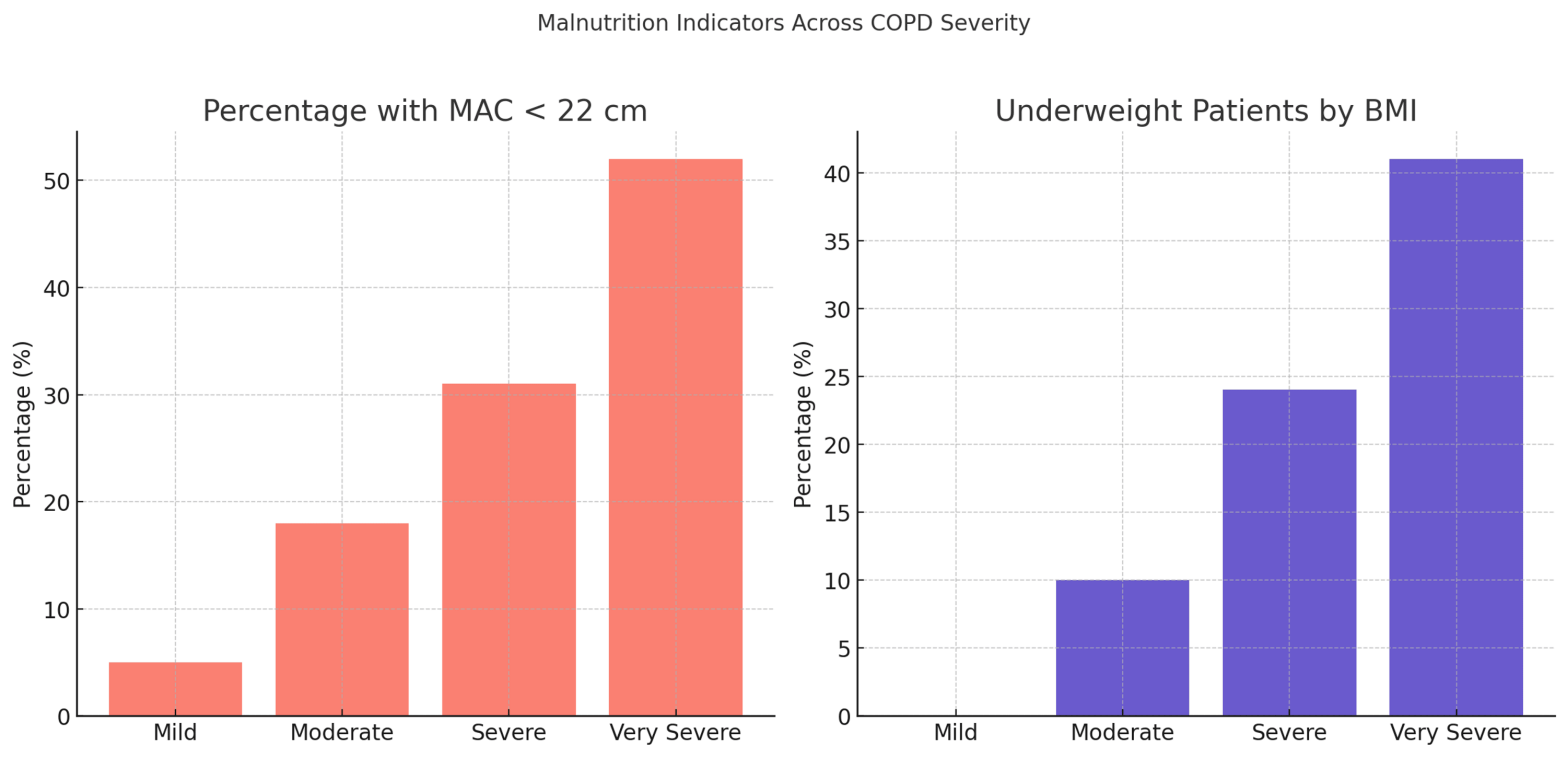
Malnutrition Indicators Across COPD Severity The percentage of patients with mid-arm circumference (MAC) < 22 cm and those classified as underweight increased significantly from mild to very severe chronic obstructive pulmonary disease (COPD) stages. These trends reflect a worsening nutritional profile in parallel with disease progression.

In Figure 2, the proportion of patients with MAC less than 22 cm - a marker of muscle mass depletion - increased markedly with disease severity, from 5% in the mild group to 52% in the very severe group. Similarly, the percentage of underweight individuals (BMI < 18.5 kg/m²) rose sharply from 0% in mild cases to 41% in the very severe group. These findings illustrate a consistent and significant deterioration in nutritional health as COPD severity advances, underscoring the clinical utility of both anthropometric and composite nutritional assessments in routine care.

## Discussion

This research underscores the substantial influence of nutritional status on the clinical management and pathophysiology of COPD. The observed positive correlations between post-bronchodilator FEV_1_% predicted and nutritional measures, including BMI, body weight, MAC, and MNA scores, support previous evidence linking malnutrition with accelerated disease progression and impaired pulmonary function in COPD patients. Among these, the MNA score demonstrated the strongest correlation with lung function, affirming its validity as a sensitive and reliable diagnostic tool for identifying malnutrition in this population. These findings are in agreement with prior studies conducted by Huxley et al., Sun et al., and Hsu et al., all of which identified significant correlations between MNA scores and critical clinical indicators, including BMI, ventilatory impairment, dyspnea severity, and the BODE (BMI, obstruction, dyspnea, and exercise capacity) index [7-9]. Benedik et al. also emphasized the prognostic utility of MNA, associating lower scores with increased risk of hospitalization and clinical deterioration [10].

While BMI is a commonly used metric for assessing nutritional status, its limitations are increasingly recognized. It does not account for critical alterations in body composition such as sarcopenia or central fat redistribution, particularly in COPD. Studies by Raad et al. and Gea et al. highlighted the insufficiency of BMI as a standalone measure and advocated for the inclusion of complementary indicators like MAC and MNA to enhance the comprehensiveness of nutritional evaluations [11,12]. Our study corroborates this multifactorial approach, demonstrating that patients with comparable BMI values can differ significantly in MAC and MNA scores across various stages of COPD as defined by the GOLD classification.

Furthermore, we observed that lifestyle determinants, specifically age and cumulative smoking exposure, were significantly associated with worsening COPD severity and nutritional decline. This information is consistent with findings reported by Noor et al., who noted a higher prevalence of malnutrition among long-term smokers and patients in advanced stages of respiratory impairment [13]. In addition, our study documented an increased burden of cardiovascular and psychiatric comorbidities in patients with very severe COPD, supporting observations by Browning et al., who linked altered body composition and central adiposity to elevated cardiometabolic risk in this population [14]. Although we did not directly assess waist-to-height ratio or advanced body composition parameters, the worsening nutritional indices and rising comorbidity prevalence in our cohort indirectly support these associations.

Data visualizations, including heatmaps and parallel coordinate plots, revealed consistent multidimensional interactions between nutritional status, age, lung function, and comorbid burden. This integrated perspective mirrors the conclusions of Collins et al., who demonstrated improved outcomes in malnourished COPD patients when nutritional support was combined with structured pulmonary rehabilitation [15]. This study has several limitations. First, due to its cross-sectional design, causal relationships between COPD severity and nutritional status cannot be established. Longitudinal studies are needed to assess changes over time and determine the directionality of associations. Second, while standardized and validated tools such as the MNA, BMI, and MAC were used, BMI alone may not adequately reflect muscle mass or functional nutritional deficits, particularly in elderly or sarcopenic patients. Third, although assessments were conducted by trained clinical staff following institutional protocols, formal training sessions or inter-rater calibration procedures were not implemented, which may introduce measurement variability. Fourth, the data collection was carried out over a six-month period (January to June 2024) at a single tertiary care center in South India; therefore, the findings may have limited generalizability to other regions or healthcare settings. Finally, potential confounding variables such as smoking history, socioeconomic status, and the presence of comorbidities were not adjusted for in the correlation analysis, which could influence the observed associations.

Despite these limitations, the study provides clinically relevant insights into the relationship between pulmonary function and nutritional status in COPD, highlighting the need for routine nutritional screening and multidisciplinary care approaches.

Hence, the findings of this study emphasize the importance of incorporating routine nutritional assessment into the standard care framework for patients with COPD. Tools such as the MNA and MAC provide accessible and informative means of identifying patients at nutritional risk. A multidisciplinary care model that integrates pulmonary rehabilitation with tailored nutritional and psychosocial support may significantly improve clinical outcomes, reduce exacerbation frequency, and enhance quality of life for individuals living with COPD.

## Conclusions

This study demonstrates a significant positive correlation between nutritional status and lung function in patients with COPD, with nutritional parameters such as BMI, MNA score, and MAC showing a progressive decline alongside increasing disease severity. Additionally, a higher burden of comorbidities was noted in more advanced stages of COPD. These findings underscore the importance of incorporating nutritional screening tools like the MNA and MAC into routine COPD evaluations to enable early detection of malnutrition and timely intervention.

From a clinical standpoint, integrating nutritional support into multidisciplinary COPD care can aid in slowing disease progression, improving functional capacity, and enhancing quality of life. Future studies should explore the longitudinal impact of targeted nutritional interventions on disease outcomes and healthcare utilization in COPD populations. Establishing standardized protocols for nutritional assessment and incorporating them into primary and specialty care pathways may further strengthen COPD management strategies.
